# Alignment-Free Sensing Module for Absolute and Incremental Lines in Linear Positioning System Based on Tunneling-Magnetoresistance Sensors

**DOI:** 10.3390/s21124137

**Published:** 2021-06-16

**Authors:** Chia-Chang Lee, Yu-Shen Yen, Chih-Huang Lai

**Affiliations:** Department of Materials Science and Engineering, National Tsing Hua University, Hsinchu 30013, Taiwan; s104031577@m104.nthu.edu.tw (C.-C.L.); oscar811002@gmail.com (Y.-S.Y.)

**Keywords:** TMR sensor, positioning system, absolute and incremental integrated system

## Abstract

An alignment-free sensing module for the positioning system based on tunneling magnetoresistive (TMR) sensors with an absolute-incremental-integrated scale is demonstrated. The sensors of the proposed system for both lines consist of identical layer stacks; therefore, all sensors can be fabricated in identical processes from thin film deposition to device patterning on a single substrate. Consequently, the relative position of the sensors can be predefined at the lithography stage and the alignment error between sensors caused by the manual installation is completely eliminated. Different from the existing sensing scheme for incremental lines, we proposed to utilize the magnetic tunnel junctions with a perpendicular anisotropy reference layer and an in-plane anisotropy sensing layer. The sensors are placed parallel to the scale plane with magnetization of the sensing layer in the plane, which show the capability of polarity detection for the absolute line and reveal sinusoidal output signal for the incremental line. Furthermore, due to the large signal of TMR, the working distance can be further improved compared with conventional sensors. In addition, the cost of the positioning system is expected to be lowered, since all the sensors are fabricated in the same process without extra installation. Our design may pave a new avenue for the positioning system based on a magnetic detection scheme.

## 1. Introduction

As the demands for automated manufacturing rise, the requirements for precise position control are enhanced. Linear positioning systems are often used in machine tools and robotic applications. Most of the positioning systems are based on optical or magnetic sensing principles. Although the optical positioning systems have higher resolution, the performance, such as accuracy, and the stability are strongly degraded in an unclean environment with the presence of oil and dust. In addition, higher energy consumption and cost are also concerned. In contrast, as the accuracy enhances, the magnetic positioning systems become more competitive due to their high environmental endurance. Furthermore, they require lower power consumption with lower cost and better scalability [1].

**Figure 1:**
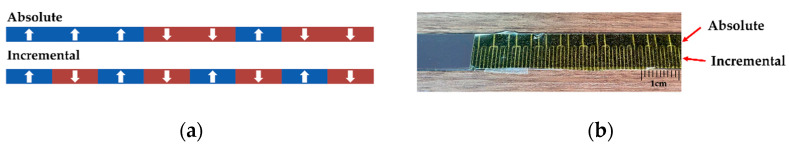
(**a**) The illustrations of incremental and absolute scales. (**b**) The integrated scale used in this work. The MFVF reveals the magnetic pattern on the scale.

Two types of magnetic positioning systems are commonly used, as shown in Figure 1a. The magnetic polarities of the absolute-type (ABS) scale are randomly altered. Each position on the ABS scale is assigned a binary code; the decoder is composed of an array of sensors with separation corresponding to the pole pitch. By reading out the polarities of each pole, the absolute position can be obtained by referring the signal to the assigned binary code [2]. The advantage of the ABS-type encoder is that the absolute position can be known immediately without initialization when rebooting the system [3]. However, the position resolution is limited by the pole pitch of the scale. In order to know the displacement distance even smaller than the pole pitch, the incremental-type (INC) is applied. The magnetic pattern of the incremental-type (INC) scale is magnetized with alternate polarities. As the sensor goes through the INC scale, the dual Wheatstone bridges in the sensor detect the magnetic flux and generate two analog sinusoidal signals with 90-degree phase shift. With the measured sine/cosine waves, the circular Lissajous curve can be created by plotting the magnitude of the sine wave on the y-axis and the cosine wave on the x-axis [4]. By dividing the Lissajous circle into many small segments, so-called the interpolation techniques, with readout IC, a displacement much smaller than the pole pitch can be detected [5,6]. However, since it only detects the relative displacement, when the system is rebooted, it always takes time to travel back to the starting point for the initialization, which increases the time cost in the manufacturing industry. To obtain the precise displacement without initialization, both scales needs to be used simultaneously. In this work, we use the integrated scale, in which both INC and ABS scales (lines) are magnetized in the perpendicular direction separately, located on the same magnetic matrix. Figure 1b shows the integrated scale with 1mm pole pitch used in this work. The magnetic pattern of both lines can be observed through the magnetic field viewing film (MFVF).

To detect the position, different magnetic sensors are used for individual lines. Typically, multiple numbers of digital Hall sensor are used to detect the N and S poles for the ABS line, and various magnetoresistance (MR) sensors, including anisotropic magnetoresistance (AMR), giant magnetoresistance (GMR), and tunneling magnetoresistance (TMR) sensors, are used to detect the angular change of the field and generate the sinusoidal output signal for the INC line due to their larger signals compared with Hall sensors [7,8,9]. However, the good alignment between sensors needs to be considered, which is difficult to be achieved with surface mount assembly process and installation. For alignment between the sensors in ABS, if one of the sensors is slightly offset from the designated position, the non-synchronized ABS position information causes the misreading of the absolute positional code, leading to a false interpretation. On the other hand, if the misalignment occurs between sensors for ABS and INC, it takes efforts to compensate the misalignment through signal processing, which is not an efficient way for the commercial products. 

To solve the misalignment issues among sensors, and to reach both of the output requirements for ABS and INC simultaneously, we propose to use the TMR sensors, composed of magnetic tunneling junctions (MTJs), for both ABS and INC lines, in which MTJs are composed of a reference layer (RL) with perpendicular magnetic anisotropy (PMA) and a sensing layer (SL) with the magnetization along the in-plane direction. The TMR sensors consisting of MTJs have been used for INC lines [10]. However, these MTJs possess the magnetization of RL and SL, both along the in-plane direction, which cannot be used for the ABS and INC detection simultaneously, as discussed in the next section. Therefore, in the existing design, TMR sensors are used for INC lines only, and extra Hall sensors are needed for ABS lines. In our design, we use the identical film stack for all sensors, so the same layer structure can be deposited on a single substrate, and the multiple sensors for both ABS and INC lines can be patterned and etched in an identical manufacturing process on the same substrate. Consequently, the cost is expected to be lower. Most importantly, the relative positions of the sensors are well-defined at the lithography stage; therefore, the feasibility of an alignment-free sensor module with easy installation is demonstrated.

## 2. Design of the Sensors for Integrated System

### 2.1. Existing System

**Figure 2:**
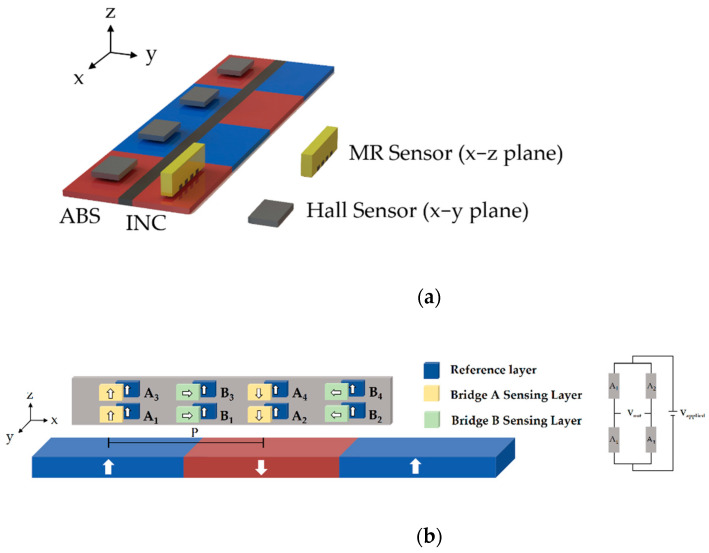
(**a**) The existing integrated system consisting of individual Hall and MR sensors. (**b**) The illustration for the magnetization directions of sensing layer (SL) and reference layer (RL) in the existing dual Wheatstone bridge TMR sensors for INC line placed in x–z plane. The schematic diagram for the Wheatstone bridge is also shown.

Figure 2a shows the setup for the ABS and INC integrated system in the existing products. For the INC line, in order to compensate the output fluctuation caused by temperature drift, a Wheatstone bridge configuration is needed for the INC sensor, typically composed of MR sensors, such as AMR or TMR sensors [10,11], in order to have a larger signal-to-noise ratio compared with Hall sensors. Ideally, the fields generated by the INC scale have two sinusoidal components, H_x_ and H_z_, which have identical field amplitude but with 90° phase difference. Therefore, the field vectors can be expressed as:

(1) $$H_{x}\;=\;H_{\max}\;\times \;\sin(x\pi /P)\;\hat{x};H_{z}\;=\;H_{\max}\;\times \;\cos(x\pi /P)\;\hat{z}$$

where H_max_ is the maximum amplitude of the field, x is the position, and P is the pole pitch. Thus, the total field experienced by the sensing element can be expressed as:

(2) $$H_{\mathrm{total}}\;=\;H_{x}\;+\;H_{z}\;=\;H_{\max}\;\times \;\sin(x\pi /P)\;\hat{x}\;+\;H_{\max}\;\times \;\cos(x\pi /P)\;\hat{z}$$

Assuming xπ/P = θ, as the sensor moves, the field experienced by sensors is equivalent to a constant amplitude of H with a varying angle θ. Therefore, if the MR sensor is placed with the magnetization of sensing and reference layers being aligned in an x–z plane (See Figure 2b), then the field provided from the scale leads to the magnetization rotation of the sensing layer and generates the desired sinusoidal output [12]. When applying the Wheatstone bridge configuration, if all the sensing elements respond identically to an applied field, then the bridge output becomes null. To make the bridge operational, in most of the applications, pinning direction of the elements in the bridge should be opposite, introducing a phase difference between elements, which usually requires additional efforts [8,13]. However, in this magnetic scale detection, the phase difference is established by the spatial separation of the elements. Therefore, the pinning directions of each element are identical. Figure 2b shows the magnetization directions of each MTJ in the existing system with TMR dual Wheatstone bridge sensors for the INC line. In this Wheatstone bridge configuration, A_1_ and A_3_ are in phase, and their resistance changes identically, while A_2_ and A_4_ are 180° phase, shifted from A_1_ and A_3_ in order to have maximum sensitivity [14]. In a single bridge, the MTJs are placed with distance P apart, making the experienced fields always 180° opposite. On the other hand, the two bridges are 90° phase shifted (0.5 P apart) to each other in order to create sine/cosine output. The magnetization of the sensing layer rotates with the angle of the external field at a different position, resulting in the desired sinusoidal outputs. On the other hand, for the ABS line, several Hall sensors are used for detecting the polarity of the poles in a perpendicularly magnetized scale. However, since multiple sensors are used in the system, the installation and the alignment between sensors are critical. For the sensors in the ABS line, the distance between sensors needs to be well controlled to be the same as the pole pitch P, or the non-synchronized ABS position information occurs. Likewise, it is also important for the alignment between sensors in INC and ABS lines. Although the misalignment can be adjusted through signal processing, if the amount of the misalignment varies, it is quite challenging to compensate the misalignment one by one. Therefore, as the number of sensors increases, the installation and the alignment become more complex. A possible solution is to integrate all the required sensors on a single substrate. However, it is quite difficult because the existing sensors for two lines are different. Most importantly, as discussed previously, the INC sensors based on angular dependence need to be placed in an x–z plane, making it impossible to integrate all the sensors on the same wafer level to sense ABS and INC lines simultaneously. 

### 2.2. Proposed System

**Figure 3:**
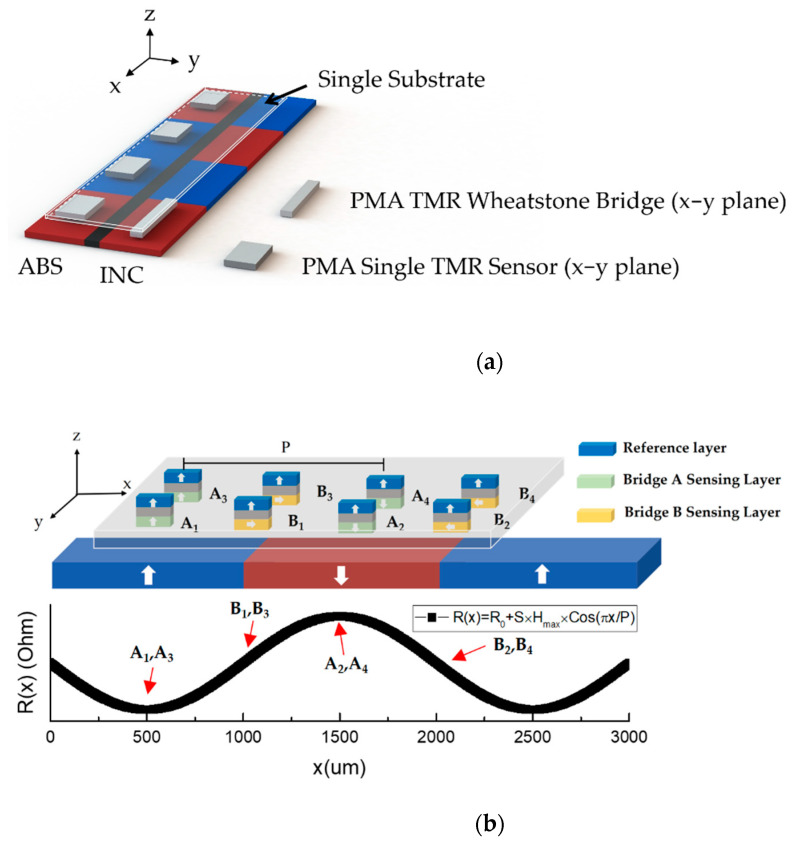
(**a**) The proposed integrated system consisting of identical TMR sensors on a single substrate. (**b**) The illustration for the magnetization directions of SL and RL in the TMR sensor for the INC line placed in the x–y plane, and the corresponding resistance for each MTJ at different positions.

In order to solve the installation and alignment problems, a new sensing scheme is proposed, as shown in Figure 3a. All the required sensors are replaced by the identical MTJs. The sensors for the ABS line consist of an array of MTJs instead of Hall sensors. The MTJs for INC are arranged into a Wheatstone bridge as the conventional system. Note that all the sensors have an identical layer stack so they can be patterned on the same wafer with the same fabrication process. Since the positions of the sensors are well-defined at the photolithograph and patterning stage, the alignment error is totally eliminated and the installation procedure is strongly simplified because all the sensors are integrated on a single substrate. To achieve this purpose, all sensors are required to be placed in the x–y plane. Therefore, all the sensor planes are parallel to the scale plane, as indicated in Figure 3a. Note that, if we used the same layer structure as the existing system, where the magnetization of RL and SL are both aligned in the x–y plane, the output signal cannot be a desired sinusoidal wave. Therefore, we propose to design our magnetization of RL along the direction perpendicular to x–y plane, and magnetization of SL aligned in the x–y plane, so-called cross-anisotropy. Unlike the existing system for INC scale, which detects both the x and z field, in our proposed system, sensors detect mainly the magnitude of z-field H_z_. Because our RL possesses strong perpendicular anisotropy, any variations of in-plane field, including x field, would not significantly change resistance. As mentioned previously, the perpendicularly magnetized INC scale exhibits field profile with a sinusoidal wave in the z direction. The intensity of H_z_ as a function of position (x) can be written as:

H_z_(x) = H_max_ × cos(xπ/P) (3)

Because of the presence of cross-anisotropy between RL and SL magnetization, the variations of resistance with the z field, the so-called RH transfer curve, should be a linear relationship [15], and can be expressed as the following:

R(H_z_) = S × H_z_ +R_0_ (4)

where S is the sensitivity, H_z_ is the applied field intensity along the z direction, and R_0_ is the resistance at zero field. Therefore, by combining (3) and (4), we can get:

R(x) = S × H_max_ × cos(xπ/P) + R_0_ (5)

As a result, the magnetization of the SL would be changed according to the magnitude of H_z_, and the sensor output would reflect the experienced H_z_ sinusoidal field profile as expressed in (5). Therefore, a sinusoidal resistance change as a function of position is obtained, which achieves the purpose of positioning the system. Figure 3b shows the working principle of our proposed INC sensor. A_1_, A_3_ and A_2_, A_4_ are separated with distance P in order to have 180° phase difference, similar to the existing system for INC sensors. Furthermore, with the linear transfer curve with respect to H_z_, the polarity detection of the perpendicularly magnetized ABS line can also be obtained by determining if the measured resistance is larger (noted as 1) or smaller (noted as 0) than the threshold resistance. 

## 3. Materials and Methods

The MTJ film was deposited by using Applied Materials Endura Clover PVD System on an 8 inch wafer at a background pressure of 2.4 × 10^−9^ torr. The layer structure for the sensor was as follows: Ta(3)/Ru(20)/Ta(1)/Pt(1)/[Co(0.75)/Pt(0.3)]_3_/Co(0.75)/Ru(0.9)/[Co(0.5)/Pt(0.3)]_2_/Co(0.6)/Ta(0.5)/CoFeB(0.85)/MgO(2)/CoFeB(t)/Ta(5)/Ru(3)/Ta(10) (the numbers represent the layer thickness in nm). Ta/Ru/Ta was used as the bottom and top electrode. The combination of CoFeB/MgO/CoFeB was selected to have a high TMR ratio due to the coherent tunneling [16]. The thickness of top CoFeB was tuned to adjust the out-of-plane anisotropy H_k_ of the sensing layer, which needed to be a moderate magnitude to fit the field range of the scale while retaining the sensitivity. Co/Pt multilayers were introduced to increase the H_c_ of RL. The synthetic anti-ferromagnet (SAF) was also applied to enhance the PMA and reduce the stray field from RL acting on SL. After the deposition, the annealing process at 350 °C for 1 h was applied to build the interfacial anisotropy between MgO and CoFeB, which caused the bottom CoFeB to become PMA and reduced the in-plane anisotropy of the top CoFeB [17,18]. The MR ratio was also enhanced due to the crystallization of CoFeB and MgO during the annealing process [19]. To demonstrate the feasibility, a single MTJ and a Wheatstone bridge were patterned for ABS and INC lines, respectively, by ion beam etching (IBE). The size of the MTJs were 15 µm × 15 µm. The magnetic property was measured by vibrating sample magnetometer (VSM) and superconducting quantum interference device (SQUID). The MR measurements were done with a 2-point probe with out-of-plane field applied. After checking the magnetic property and MR response, the devices were wire-bonded and packaged for the scale measurement. The integrated magnetic scale with 1 mm pole pitch was placed on the platform driven by a linear motor. The sensors were fixed above the scale with moderate air gaps. The output signals were detected while the relative displacement between the scale and the sensor presented.

## 4. Results and Discussion

**Figure 4:**
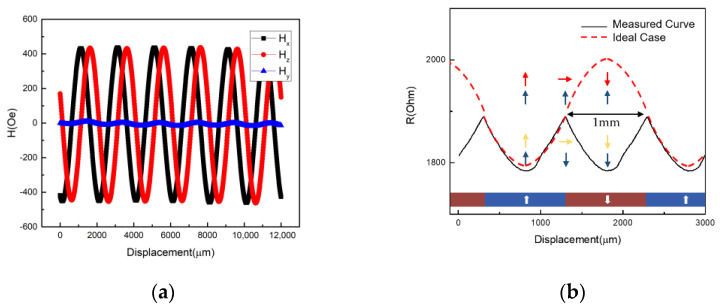
(**a**) The field profile of the INC line measured by 3-axis Hall sensor. (**b**) The output signal for the MTJ with RL switched during the scale measurement.

In our proposed magnetic scale-based positioning system, several factors need to be carefully considered according to the field profiles of the scale. Figure 4a shows the field profile of the INC line, with perpendicular magnetization measured by a 3-axis Hall sensor with air gap = 0.1 mm. Both H_x_ and H_z_ exhibit sinusoidal behavior with identical magnitude and a 90° phase difference. The H_y_ is quite small compared with H_x_ and H_z_. To achieve a high-performance positioning sensor, the following magnetic properties need to be well-controlled. First of all, the coercivity (H_c_) of the RL needs to be larger than H_z,max_, which prevents the RL from switching during the measurement. It is worth mentioning that, even if the H_c_ of RL is larger than H_z,max_, with the assistance of the H_x_, the RL may still be switched based on the Stoner–Wohlfarth astroid [20]. Either the low H_c_ or H_k_ would make the RL switched, which leads to an undesirable output signal. Here we show one example in Figure 4b, in which the RL is only composed of Pt/Co multilayer without the SAF structure, so that the H_c_ of RL is reduced to only 500 Oe and H_k_ = 2 k Oe. Figure 4b shows the measurement results with pole pitch equal to 1 mm for the INC scale. Since our sensor possesses cross-anisotropy, if the magnetization of RL is firmly pinned, the linear RH response should lead to the periodicity of the output signal equal to 2P, as shown in Figure 3b (also shown in Figure 4b as the red dash line). The arrows in Figure 4b show the magnetization directions of the pinned and sensing layer for the sensors with strong PMA (ideal case) and weak PMA (H_k_ = 2 k Oe, measured curve), respectively. The magnetization of the sensing layers rotates identically in both cases; however, when the perpendicular anisotropy of RL is not strong enough, the magnetization of RL is switched with the assistance of H_x_ during the measurement, resulting in sign change of dR/dx and a triangle-like sharp peak with a wrong periodicity (~1 mm). Therefore, the strong PMA is essential to suppress the RL tilting caused by the presence of H_x_.

**Figure 5:**
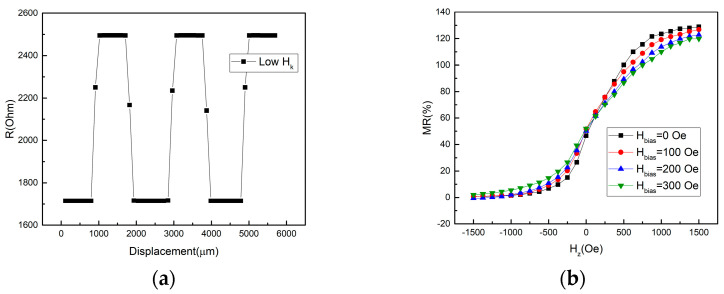
(**a**) The square output caused by the small H_c_/H_k_ of SL. (**b**) The RH curves influenced by different magnitudes of bias field.

The control of the anisotropy field (H_k_) of the SL is also critical. If the SL exhibits a large in-plane anisotropy, a strong z-field is needed to drive the magnetization of SL to be tilted away from the x–y plane, resulting in a low sensitivity. If the anisotropy of SL is too small or even exhibits PMA, the SL will be saturated easily during the position sensing, leading to a square-like output instead of a sinusoidal output, as shown in Figure 5a. Although the disturbance of H_x_ on RL can be suppressed by utilizing magnetic layers with strong PMA, the disturbance of H_x_ also exerts on the SL. Due to the H_x_ presence, it behaves as the stabilizing field for SL magnetization aligned in the x–y plane; therefore, the tilting of SL magnetization away from the x–y plane depends on the magnitude of H_x_ field. Since H_x_ varies with position, the saturation field of SL along the z direction and sensitivity (S) change accordingly. Figure 5b shows the out-of-plane RH curves with different magnitudes of in-plane bias fields. Ideally, according to Equation (5), the sensitivity (S) is a constant. However, the position-dependent x field, behaving as the bias field, may result in the variations of sensitivity [21]. As a result, the sensitivity changes as the sensor moves, which leads to a nonlinear transfer curve with respect to out-of-plane field, which may not precisely reflect the sinusoidal field profile of the scale. In fact, the sensitivity affected by the presence of the orthogonal field to the sensing direction, so-called the cross-field effect, is also observed in other types of MR sensors [22,23]. Its effects on the position accuracy will be discussed later. 

**Figure 6:**
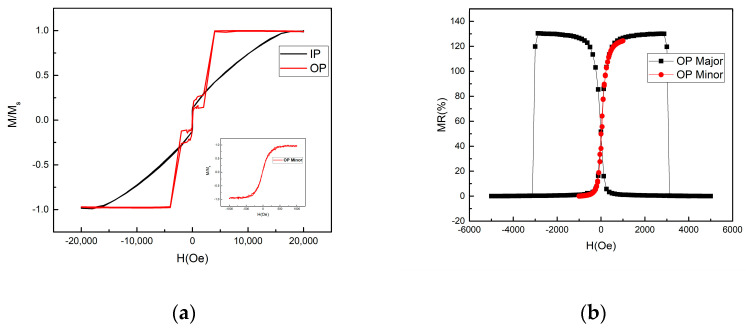
(**a**) The in-plane (IP) and out-of-plane (OP) hysteresis loops of the full-stack MTJ film after annealing. The inset shows the out-of-plane minor loop. The lower saturation magnetization in the IP loop is caused by the unsaturated SAF under 1.5 T in-plane magnetic field. (**b**) The RH transfer curves of the MTJ. The black (red) curve is the major (minor) RH curves.

The magnetic property of the full structure MTJ after annealing at 350 °C is shown in Figure 6a. The SAF pinning layer significantly enhances the coercivity of the reference layer, and the pinning field of the SAF is around 2500 Oe. The in-plane curve shows that the H_k_ of the CoPt–SAF pinned layer is around 20 kOe, indicating that the SAF structure indeed provides strong PMA. As a result, a robust RL is obtained. The minor loop shown in the inset of Figure 6a reveals a linear response, indicating that the sensing layer rotates coherently with the field applied along the out-of-plane direction. The optimized thickness of the CoFeB sensing layer is 1.6 nm. At t = 1.6 nm, the H_k_ of the SL is round 250 Oe. Figure 6b shows the RH transfer curves with an out-of-plane applied field for the single MTJ device. The MR is 130% and the reference layer magnetization is fixed within the dynamic range of the sensing layer, resulting in a high effective MR response. The minor loop of the RH curve shows the desired linearity and low hysteresis. The transfer curve of the sensing layer is symmetric to the zero field, indicating that the stray field from the reference layer is significantly suppressed as a result of the compensated magnetizations in SAF [24]. The H_k_ of the SL is slightly increased to 500 Oe during the etching process.

After checking the magnetic and electrical properties of the single device, we patterned the single MTJ for the ABS line and the Wheatstone bridge for the INC line. For the ABS line, since it only needs to detect the polarity, there is no need to form a bridge configuration. In contrast, Wheatstone bridges are required in the INC line since the resistance fluctuates with the ambient temperature, resulting in a fluctuating signal and influencing the position accuracy.

**Figure 7:**
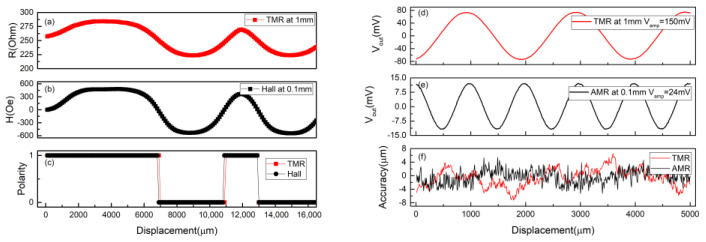
The measurement results of the ABS line measured by (**a**) TMR sensor and (**b**) commercial Hall sensor. (**c**) The corresponding polarities of the sensors. The measurement results of the INC line measured by (**d**) TMR sensor and (**e**) commercial AMR sensor. (**f**) The accuracy comparison of the sensors. Only single bridge outputs for INC are shown here.

The scale measurement is performed with the sensors placed in the x–y plane above the integrated scale shown in Figure 1b. The applied voltage for the MTJ sensors is 1V. Figure 7a shows MTJ output for the ABS line at air gap = 1 mm. The output signal shows clear high and low resistance states corresponding to the N and S poles, respectively. To further evaluate the ABS sensor performance, the commercial Hall sensor is also used to measure the same range but with air gap = 0.1 mm, as shown in Figure 7b. The measured profile of our sensor highly matches the Hall sensor. Figure 7c shows the corresponding polarity. The digital signal is converted from the measured analog signals. The results show that the TMR sensor does match the Halls sensor, capable for the ABS sensing, but with a higher gap tolerance. Figure 7d show the INC measurements of one of the TMR Wheatstone bridges with sensor at gap = 1.0 mm. The bridge exhibits a sinusoidal output with high sensitivity. To make a comparison, we also benchmarked the commercial INC sensor based on AMR effect (Figure 7e). The applied voltage for the AMR sensor is 1V. Obviously, our TMR sensor provides a six times higher signal at gap = 1.0 mm compared with the AMR sensor at 0.1 mm. We also simulated the accuracy of the INC sensor based on the dual bridge output to evaluate the performance of the sensor. The ideal position sensors reveal quadrature sinusoidal output signals while passing across the scale. From the viewpoint of positioning, the output signals of the INC sensor can be transferred by interpolation technique into position. The error between ideal and measured position is known as accuracy. The accuracy simulation is obtained as follows. First of all, the ideal sinusoidal fitting curves (V_A.ideal_ and V_B.ideal_) are obtained from the signals measured by the dual bridge (V_A.measured_ and V_B.measured_). By comparing the difference between arctan (V_A.ideal_/V_B.ideal_) and arctan (V_A.measured_/V_B.measured_), we can obtain the deviation from the ideal position [25]. The results of simulated accuracy are shown in Figure 7f. The accuracy error of TMR sensor at gap = 1 mm is within the range of ±6.5 μm, which is comparable to the AMR sensor at 0.1 mm. Although the accuracy needs to be further improved, the working distance can be much higher in our TMR sensor. The high working distance not only simplified the installation, but provided the tolerance of the vibration and impurity in the factory. The results prove that both ABS and INC lines can be detected by using the identical layer stack with sensing planes parallel to the scale plane, which makes all the sensors able to be fabricated on a single substrate. In our proposed scheme, the sensor position is well-defined and the alignment error between sensors in the integrated system is totally excluded.

Finally, we would like to discuss the possible solution for further improving the accuracy of our proposed system compared with the AMR sensor for the INC line. As illustrated previously in Figure 5b, the varied H_x_ biasing field keeps changing the sensitivity of the sensing layer, resulting in an imperfect linear transfer curve, which lowers the accuracy. In our setup, only the x-direction bias field needs to be considered, and the H_y_ from the scale can be neglected according to Figure 4a. Therefore, to suppress the changed tilting caused by a varying H_x_ bias field with the position, we may provide an additional stabilizing field along the y direction to stabilize the SL magnetization on the x–y plane. Methods such as shape anisotropy or exchange bias along the y-axis can be expected to further improve the accuracy of the INC line. 

## 5. Conclusions

In summary, we demonstrate the integrated positioning module on a single substrate to simultaneously sense the ABS and INC line. Through the crossed-anisotropy built by the PMA reference layer and in-plane sensing layer, we are able to make all the sensors in the positioning system parallel to the magnetic scale surface, allowing the sensors to be integrated on the same substrate. Since the relative position has been defined in the lithography stage, alignment error between sensors during the installation is totally excluded. In addition, we also reveal the critical requirements for the PMA of the reference layer and anisotropy field for the sensing layer. By properly tuning the magnetic properties of TMR sensors, we demonstrate a substantially increased output for the INC scale with the pole pitch of 1 mm and the air gap of 1 mm, compared to a conventional AMR sensor with the air gap of 0.1 mm. Furthermore, the output profile of a TMR sensor for the ABS line is similar to the conventional Hall sensors, but with enhanced amplitude and height tolerance. Our design may pave a new avenue for the positioning system based on the MR detection scheme. 

## Data Availability

Not applicable.
